# Candidemia in a tertiary hospital in Southern Brazil

**DOI:** 10.1590/S1678-9946202668027

**Published:** 2026-04-10

**Authors:** Leandre Carmem Wilot, Bianca dos Santos Blan, Vanice Rodrigues Poester, Cecília Bittencourt Severo, Karine Ortiz Sanchotene, Mônica Campos dos Santos, Mariana Rodrigues Trápaga, Melissa Orzechowski Xavier

**Affiliations:** 1 Universidade Federal do Rio Grande, Faculdade de Medicina, Programa de Pós-Graduação em Ciências da Saúde, Rio Grande, Rio Grande do Sul, Brazil; 2 Universidade Federal do Rio Grande, Faculdade de Medicina, Laboratório de Micologia, Rio Grande, Rio Grande do Sul, Brazil; 3 Universidade Federal do Rio Grande, Faculdade de Medicina, Hospital Universitário Dr. Miguel Riet Corrêa Jr., Rio Grande, Rio Grande do Sul, Brazil; 4 Universidade Federal de Ciências da Saúde de Porto Alegre, Porto Alegre, Rio Grande do Sul, Brazil

**Keywords:** *Candida* spp, Epidemiology, Fungemia, Blood culture, Antifungal resistance

## Abstract

Invasive bloodstream infections caused by *Candida* spp. significantly impact global health and are associated with high mortality rates. This study describes a five-year retrospective analysis conducted in a tertiary hospital with 213 beds in Rio Grande, Rio Grande do Sul State, Brazil, aiming to provide clinical-epidemiological data on candidemia and the susceptibility profile of isolates in this region. From January 2019 to December 2023, 44 patients (26 adults, 15 neonates, and three pediatric individuals) were diagnosed with candidemia at our hospital and included in this study. *Candida* species accounted for 4.5% of all sepsis-causing agents (44/971). The overall incidence was 2.2 cases per 1,000 hospitalizations; however, when stratified by hospitalization unit, this rate reached 26 per 1,000 hospitalizations in the neonatal intensive care unit (NICU), followed by 13 per 1,000 hospitalizations in the intensive care unit (ICU). The mortality rate reached 92% among ICU patients and 40% among NICU patients. *C. albicans* was the most prevalent species (57%), while *C. parapsilosis* was the most common non-*albicans* species (∼30%). Resistance to at least one of the three antifungal classes tested was detected in 13% of the isolates. This study underscores the substantial impact of candidemia in ICU settings in a tertiary hospital in Southern Brazil, emphasizing the importance of improved awareness and surveillance to enhance diagnosis, control, prevention, and monitoring of antifungal resistance, aiming to reduce incidence and mortality rates.

## INTRODUCTION

Candidemia, a bloodstream infection, predominantly affects critically ill patients and is the leading hospital-acquired invasive fungal infection (IFI)^1,2^. Risk factors include a wide range of conditions, particularly hospitalization in intensive care units (ICU), total parenteral nutrition, chemotherapy, broad-spectrum antibiotic therapy, abdominal surgery, use of medical devices (central venous catheters, intravascular lines, or prostheses), neutropenia, and extremes of age (prematurity or advanced age)^3,4^.

Although candidemia is an important healthcare-associated infection (HAI), the non-mandatory reporting of cases and diagnostic challenges mean that its true burden is not precisely known. Global estimates of candidemia incidence range from 0.33 to 6.51 episodes per 1,000 admissions, with associated mortality rates of up to 60%^2,5-8^. In Brazil, Giacomazzi *et al*.^9^ estimated approximately 29,000 new candidemia episodes per year. However, a systematic review^10^ published in 2024 identified only 16 studies, comprising 2,305 candidemia episodes, compared with an estimated 203,000 expected cases for the 2017–2023 period based on Giacomazzi's projections. This discrepancy highlights the likely underdiagnosis and underreporting of candidemia in Brazil.

*Candida albicans* is the most common species, representing approximately ∼40% of candidemia cases in Brazil^10^. Additionally, concerns have been raised regarding the increasing incidence of invasive infections caused by non-*albicans Candida* (NAC) species, including *C. parapsilosis, C. tropicalis, Nakaseomyces glabratus* (former *C. glabrata*), *Pichia kudriavzevii* (former *C. krusei*), and *Meyerozyma guilliermondii* (former *C. guilliermondii*)^11-13^. Species distribution varies according to geographic region, type of ICU (adult or neonatal), host conditions, antifungal prophylaxis, and resources available for HAI prevention^14-16^.

Given the emergence of NAC species as multidrug-resistant pathogens and their association with outbreaks, together with the high prevalence of *C. albicans* and the severity of infections caused by these organisms, the World Health Organization has classified *C. albicans, C. auris, C. parapsilosis*, and *C. tropicalis* as critical/high-priority pathogens^17^. Considering the clinical impact of candidemia and the lack of data on this IFI in Southern Brazil, we aimed to evaluate clinical-epidemiological characteristics of candidemia and the susceptibility profile of isolates over a five-year period in a tertiary hospital in the Southern region of Rio Grande do Sul State, Brazil.

### Ethics

This study was approved by the Research Ethics Committee of Universidade Federal do Rio Grande, process Nº 53413821.8.0000.5324.

## MATERIALS AND METHODS

A retrospective study was conducted at Hospital Universitário Dr. Miguel Riet Correa Jr. (HU), affiliated with Universidade Federal do Rio Grande (FURG) and Empresa Brasileira de Servicos Hospitalares (EBSERH) (Rio Grande, Rio Grande do Sul State, Brazil). HU-FURG/EBSERH is fully integrated into the Brazilian Unified Health System (SUS – Sistema Unico de Saude) and has 213 beds, including six ICU beds and 10 neonatal ICU (NICU) beds. The hospital is a referral center for medium- and high-complexity care for over 20 municipalities within the 7^th^ Regional Health Coordination in Southern Brazil.

All patients with positive peripheral blood cultures for *Candida* spp. from January 2019 to December 2023 were included in this study. Patients with a new episode of candidemia (defined as a positive peripheral blood culture for *Candida* spp. occurring 30 days after the initial diagnosis) were also included. Peripheral blood samples collected in BD BACTEC^™^ Plus Aerobic/F (Aero) bottles (Becton Dickinson, East Rutherford, USA) were incubated using the BD BACTEC^™^ FX 40 automated system (BD Diagnostics, Sparks, USA). Sequentially, isolates were purified in Sabouraud Dextrose Agar (KASVI, São José dos Pinhais, Brazil) and CHROMagar^™^ *Candida* (DIFCO, Michigan, USA), and identified based on protein profile using matrix-assisted laser desorption ionization–time of flight mass spectrometry (MALDI-TOF MS) (Bruker Corporation^™^, Billerica, Massachusetts, USA; database flex control version 3.4). The susceptibility profile of the clinical isolates to fluconazole (FCZ), amphotericin B (AMB), and micafungin (MICA) was evaluated according to the M27-A2 Clinical & Laboratory Standards Institute protocol^18^.

We analyzed the hospital database to obtain clinical-epidemiological data, including sex, age, hospital unit, comorbidities, length of hospitalization prior to candidemia diagnosis, *Candida* species, use of broad-spectrum antibiotics and/or steroids, antifungal therapy, mechanical ventilation, presence of central venous access, history of recent abdominal surgery, use of total parenteral nutrition, thrombocytopenia, and hospitalization outcome (death or hospital discharge). The hospital mortality rate was calculated for each evaluated unit. Moreover, for patients admitted to the NICU, gestational age (extremely preterm [< 28 weeks], very preterm [28 to < 32 weeks], moderate to late preterm [32 to < 37 weeks], and term [≥37 weeks]) and birth weight (extremely low weight [<1,000 g], very low weight [1,000 to <1,500 g], low weight [1,500 to < 2,500 g] and appropriate weight [≥2,500 g]) were evaluated.

Data were analyzed using SPSS 24.0 (SPSS, Chicago, IL, USA) using descriptive frequency and measures of central tendency. Overall and annual prevalence rates were calculated, and the incidence of candidemia per 1,000 hospitalizations (overall and stratified by hospitalization unit) was estimated. The impact of risk factors, treatment, and causative species on clinical outcomes (death or hospital discharge) among NICU patients was analyzed using the chi-square test. Statistical significance was set to *p* < 0.05.

## RESULTS

**Figure 1:**
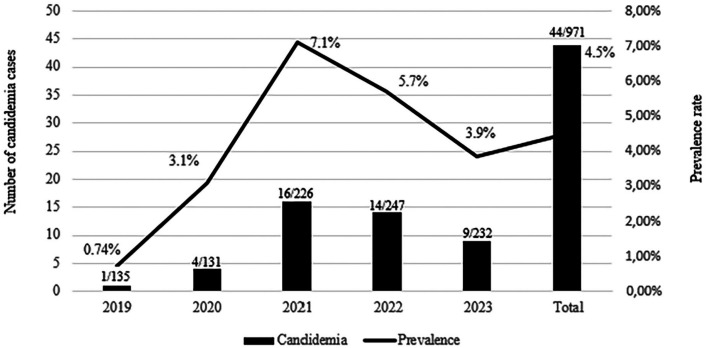
Non-linear annual distribution of candidemia cases (number of cases and prevalence rate) diagnosed during the five-year study conducted at Hospital Universitario Dr. Miguel Riet Correa Jr.

During the five-year study period, 44 patients were diagnosed with candidemia at HU-FURG/EBSERH. Overall, *Candida* species accounted for 4.5% (44/971) of all sepsis-causing agents identified in our hospital. The median number of candidemia cases was 8.8 cases/year, ranging from one to 16 cases, corresponding to prevalence rates ranging from 0.74% (1/135) to 7.1% (16/226) according to the study year (Figure 1).

Of the 44 candidemia cases, 26 occurred in adults, 15 in neonates, and three in pediatric patients. Regarding hospitalization units, 15 (34%) patients were admitted to the NICU, 14 (32%) to the adult ward, 12 (27%) to the ICU, two (4.5%) to the pediatric ward, and one (2.3%) to the pediatric ICU (Table 1).

**Table 1:** Clinical-epidemiological data of 44 patients diagnosed with candidemia at Hospital Universitario Dr. Miguel Riet Correa Jr., 2019–2023.

		Total (n=44)	Hospital care units		
			Adults ICU+Clinic (n=26)	Neonatal ICU (n=15)	Pediatric (n=3)
**Sex**		**n (%)**	**n (%)**	**n (%)**	**n (%)**
	Male	23 (52.3)	14 (53.8)	8 (53.3)	1 (33.3)
	Female	21 (47.7)	12 (46.2)	7 (46.7)	2 (66.7)
**Risk/Clinical factors**		**n (%)**	**n (%)**	**n (%)**	**n (%)**
	Use of broad-spectrum antibiotics	44 (100)	26 (100)	15(100)	3 (100)
	Central venous access	42 (95.5)	24 (92.3)	15 (100)	3 (100)
	Mechanical ventilation	28 (63.6)	13 (50)	14 (93.3)	1 (33.3)
	Use of steroids	26 (59.1)	20 (76.9)	6 (40)	0
	Use of total parenteral nutrition	22 (50)	5 (11.5)	15 (100)	2 (66.7)
	Recent abdominal surgery	17 (38.6)	11 (42.3)	4 (26.7)	2 (66.7)
	Thrombocytopenia	21 (47.7)	10 (38.5)	10 (66.7)	1 (33.3)
	Recurrent fever	11 (25)	7 (26.9)	3 (20)	1 (33.3)
Antifungal treatment*		**n (%)**	**n (%)**	**n (%)**	**n (%)**
	Yes	29 (66)	13 (50)	15 (100)	1 (33.3)
	No	13 (29.5)	12 (46)	0	1 (33.3)
	Missing	2 (4.5)	1 (4)	0	1 (33.3)
**Duration of antifungal treatment**		**n (%)**	**n (%)**	**n (%)**	**n (%)**
	< 14 days	10 (34.5)	7 (53.8)	3 (20)	0
	≥ 14 days	19 (65.5)	6 (46.2)	12 (80)	1 (100)
**Causative specie**		**n (%)**	**n (%)**	**n (%)**	**n (%)**
	*Candida albicans*	25 (56.8)	16 (61.5)	9 (60)	0
	*C. parapsilosis*	13 (29.5)	4 (15.4)	6 (40)	3 (100)
	*Nakaseomyces glabratus*	1 (2.3)	1 (3.85)	0	0
	*Meyerozyma guilliermondii*	1 (2.3)	1 (3.85)	0	0
	*C. tropicalis*	1 (2.3)	1 (3.85)	0	0
	*Pichia kudriavzevii*	1 (2.3)	1 (3.85)	0	0
	*C. duobushaemulonii*	1 (2.3)	1 (3.85)	0	0
	*C.* non*-albicans* (unidentified)	1 (2.3)	1 (3.85)	0	0
**Outcome**		**n (%)**	**n (%)**	**n (%)**	**n (%)**
	Death	22 (50)	16 (61.5)	6 (40)	0
	Hospital discharge	20 (45.5)	9 (34.5)	9 (60)	2 (66.6)
	Missing	2 (4.5)	1 (4)	0	1 (33.3)

ICU = intensive care unit;

* Treatment was administered with fluconazole and/or amphotericin B.

The total number of hospitalizations during the study period at HU-FURG/EBSERH was 19,802, resulting in an estimated overall incidence of candidemia of 2.2 cases per 1,000 hospitalizations. When stratified by hospital unit, the incidence was considerably higher in the NICU, with 26 cases per 1,000 hospitalizations, followed by the ICU, with 13 cases per 1,000 hospitalizations. In pediatric and adult wards, the incidence was one case per 1,000 hospitalizations.

### Candidemia in adult patients

Among adults diagnosed with candidemia (n=26) in the ICU and ward, the median age was 46 years (range: 16-83 years), and 53% (14/26) were male. All patients had at least one comorbidity, including kidney failure (50%; 13/26), systemic arterial hypertension (38%; 10/26), neoplasia (31%; 8/26), COVID-19 (23%; 6/26), diabetes mellitus, human immunodeficiency virus (HIV) infection and pulmonary tuberculosis (19% each; 5/26), anemia, chronic obstructive pulmonary disease and cerebral ischemia (15% each; 4/26), autoimmune diseases and hepatitis C virus infection (11% each; 3/26), and thyroid dysfunction, kidney transplant, and neurotoxoplasmosis (4% each; 1/26). Additionally, these patients were exposed to several risk factors (Table 1).

The mean duration of hospitalization prior to candidemia diagnosis was 21 days (range: 4–127 days). The average length of hospitalization between diagnosis and clinical outcome was 29 days (range: 17–169 days). *C. albicans* was the etiological agent in 62% (16/26) of cases, followed by *C. parapsilosis* (15%; 4/26). *N. glabratus* (formerly *C. glabrata*), *M. guilliermondii* (former *C. guilliermondii*), *C. tropicalis, P. kudriavzevii* (formerly *C. krusei*), and *C. duobushaemulonii* accounted for one case each (4%). One case was caused by a non-*albicans* species that could not be identified by MALDI-ToF and was therefore reported as *Candida* sp. (non-*albicans*) (Table 1).

Data on antifungal treatment and outcomes were available for 96% (25/26) of adult patients. Treatment with FCZ and/or AMB was administered to 13 patients; eight died before treatment initiation, one was transferred to another hospital, and in the remaining four cases, antifungal treatment was not prescribed (Table 1). The overall mortality rate among adult patients with candidemia was 64% (16/25), with a mean time of death of 22 days after diagnosis (range: 1–169). Mortality was 92% (11/12) among ICU patients and 36% (5/14) among patients in the adult ward (*p*=0.02). Among deceased patients, 81% (13/16) died within 30 days after diagnosis. No differences in mortality were observed among adult patients according to the etiological agent (*Candida* species). Regarding risk factors, mechanical ventilation and thrombocytopenia were associated with mortality (*p*=0.002).

### Candidemia in neonate patients

All neonates with candidemia (n=15) were admitted to the NICU, with a median age of 46 days (range: 14-150 days old); 53% (8/15) were male (Table 1). All patients had at least one comorbidity, including respiratory distress syndrome (53%; 8/15), neonatal jaundice (13%; 2/15), renal injury, gastroschisis, esophageal atresia, necrotizing enterocolitis, and hypothyroidism (7% each; 1/15), and were exposed to well-established risk factors for candidemia, including extreme prematurity and extremely low birth weight (Tables 1 and 2).

**Table 2:** Impact of risk factors, treatment, and causative species on clinical outcomes among 15 patients with candidemia in the neonatal intensive care unit at Hospital Universitario Dr. Miguel Riet Correa Jr., 2019–2023.

		Death (n=6)	Hospital discharge (n=9)	*p*-value*
*Candida* **species**		**n (%)**	**n (%)**	0.287
	*C. albicans*	5 (55.5)	4 (44.5)	
	*C. parapsilosis*	1 (16.7)	5 (83.3)	
**Duration of antifungal treatment**		**n (%)**	**n (%)**	0.024*
	< 14 days	3 (100)	0	
	≥ 14 days	3 (25)	9 (75)	
**Thrombocytopenia**		**n (%)**	**n (%)**	0.469
	Yes	5 (50)	5 (50)	
	No	1 (20)	4 (80)	
**Gestational age**		**n (%)**	**n (%)**	0.549
	Extremely premature (< 28 weeks)	4 (50)	4 (50)	
	Very premature (28 to < 32 weeks)	0	3 (100)	
	Moderate to late premature (32 to < 37 weeks)	2 (66.6)	1 (33.4)	
	Term (≥ 37 weeks)	0	1 (100)	
**Weight at birth**		**n (%)**	**n (%)**	0.912
	Extremely low weight (< 1,000 g)	4 (44.4)	5 (55.6)	
	Very low weight (≥1,000 to < 1,500 g)	1 (33.3)	2 (66.7)	
	Low weight (≥1,500 to < 2,500 g)	1 (33.3)	2 (66.7)	

* Chi-square test. Statistical significance was set at *p* < 0.05.

The mean duration of hospitalization prior to candidemia diagnosis was 21 days (range: 9–117 days). The average length of hospitalization between diagnosis and clinical outcome was 40 days (range: 0–117 days). *C. albicans* was the causative agent in 60% (9/15) of cases, while *C. parapsilosis* accounted for the remaining 40% (6/15) (Table 1). All patients received antifungal treatment with FCZ or AMB for a mean duration of 16 days (range: 6–40 days). The outcome of patients was death in 40% (6/15) of cases, with a mean time from diagnosis to death of seven days (range: 0–13). Among deceased patients, 50% (3/6) died within 30 days after diagnosis. According to the causative species, the mortality rate was 55.5% in *C. albicans* infections (5/9) and 16.7% in *C. parapsilosis* infections (1/6) (Tables 1 and 2).

### Candidemia in pediatric patients

Among the three pediatric patients diagnosed with candidemia, admitted to the pediatric ICU or pediatric ward and aged six months, four years, and 10 years, most were female (66%; 2/3). Predisposing factors included broad-spectrum antibiotics (3/3), central venous access (3/3), recent abdominal surgery (2/3), total parenteral nutrition (2/3), and mechanical ventilation (1/3) (Table 1).

The interval between hospitalization and candidemia diagnosis was 47 days (range: 19–79 days), and *C. parapsilosis* was the etiological agent of all cases. The four-year-old patient showed clinical improvement and recovered from the infection, although treatment details were not available in the hospital database. Another patient recovered and was discharged after treatment with AMB and FCZ for 14 days, while one patient was lost to follow-up after being transferred to another hospital (Table 1).

### Antifungal susceptibility

Antifungal susceptibility testing was performed on 39 of the 44 *Candida* isolates, revealing resistance to at least one antifungal agent in 13% (n=5) of cases. Susceptible dose-dependent (SDD) responses to FCZ were observed in three isolates (one *C. albicans*, one *C. parapsilosis*, and one *P. kudriavzevii*, an intrinsically resistant species). Resistance to MICA was detected in two isolates (*C. tropicalis* and *C. parapsilosis*, the latter also resistant to FCZ), whereas resistance to AMB was observed only in a *C. duobushaemulonii* isolate. Detailed minimum inhibitory concentration (MIC) values and susceptibility profiles are presented in Table 3.

**Table 3:** Antifungal susceptibility profile of *Candida* spp. Isolates (n=39) recovered during a five-year study of bloodstream infection conducted at Hospital Universitario Dr. Miguel Riet Correa Jr., 2019–2023

Species / Antifungal	Fluconazole				Amphotericin B			Micafungin		
	MIC (µg/mL)	Resistance N (%)	ID of resistant isolates	SDD N (%)	MIC (µg/mL)	Resistance N (%)	ID of resistant isolates	MIC (µg/mL)	Resistance N (%)	ID of resistant isolates
*C. albicans* (n=21)	1->64	1 (5.0)	M 7462	2 (9.5)	0.25- 1	0	-	0.03-1.0	0	-
*C. parapsilosis* (n=12)	1- >64	1 (8.3)	M 9420	0	0.25- 1	0	-	0.03>8	1 (8.3)	M 9420
*C. tropicalis* (n=2)	2- 8	0	-	0	0.25- 1	0	-	0.03>8	1 (50)	M 10464
*Pichia kudriavzevii* (n=1)	>64	1 (100)*	M 6336	0	0.5	0	-	0.125	0	-
*M. guilliermondii* (n=1)	4	0	-	0	0.5	0	-	0.5	0	-
*Nakaseomyces glabratus* (n=1)	16	0	-	1 (100)	0.5	0	-	0.03	0	-
*C. duobushaemulloni* (n=1)	8	0	-	0	2	1 (100)	M 8268	0.06	0	-

MIC = Minimum inhibitory concentration; SDD = Susceptible-dose dependent; ID = Identification of isolates classified as resistant;

* intrinsic resistance.

## DISCUSSION

Our study is the first to describe clinical-epidemiological data on candidemia in a tertiary hospital (HU-FURG/EBSERH), a referral center for 22 municipalities in the Southernmost region of Brazil. The findings reveal the impact of this infection, especially among critically ill patients. Although the overall incidence was 2.2 cases per 1,000 hospitalizations, higher rates were observed in ICUs, reaching 26 cases per 1,000 hospitalizations in the NICU and 13 per 1,000 hospitalizations in the ICU. These rates are much higher than the globally reported ICU incidence rates, which range from 6.51 to 7.07 cases per 1,000 hospitalizations^7,8^.

All patients diagnosed with candidemia had well-established risk factors commonly reported in the scientific literature^19-21^, including use of broad-spectrum antibiotics, mechanical ventilation, total parenteral nutrition, prematurity in neonates, and central venous or umbilical catheters. Moreover, our data reinforce that thrombocytopenia is an important prognostic marker among adults in ICUs, as it was associated with mortality (*p=*0.002), as previously reported^20-22^. However, the small sample size represents a limitation of our study. Another limitation was the lack of information regarding the removal of central venous catheters and the investigation of endophthalmitis and endocarditis, particularly in cases of persistent candidemia.

Our highest prevalence occurred during the pandemic years. The increase in candidemia incidence may have been influenced by COVID-19 infections, as patients with severe COVID-19 are at increased risk of developing candidemia. These individuals are exposed to multiple risk factors, including the use of antibiotics, corticosteroids, venous catheters, and dialysis^23,24^. Additionally, hospital overcrowding during the pandemic may have contributed to the increased number of candidemia cases.

Our study revealed a 40% mortality rate among neonates who developed candidemia. This finding, coupled with the absence of antifungal prophylaxis, highlights the urgent need for preventative measures in neonatal care settings. Current guidelines recommend antifungal prophylaxis in neonatal units where the incidence of invasive fungal infections is ≥2%^25^. Notably, our incidence was 26 cases per 1,000 hospitalizations, well above this threshold. The lack of prophylaxis in this high-risk population may have contributed to the high rate of poor outcomes. These findings underscore the need to implement antifungal prophylaxis to improve survival rates and reduce complications among neonates at risk of invasive fungal infections. Furthermore, the literature^26^ emphasizes the importance of strict catheter care and hand hygiene among healthcare staff, particularly given the high incidence of *C. parapsilosis*.

Only 65% of our patients received antifungal treatment in accordance with current guideline recommendations^15,26-28^, including therapy for at least 14 days after the first negative blood culture. Moreover, mortality ranged from 40% in the NICU to 92% in the ICU, reflecting the severity of illness in these settings. These rates are similar to or higher than those reported in other Brazilian studies5,^29,30^. It is important to highlight that appropriate antifungal therapy initiated within 24 hours of shock onset reduces mortality^30^, as does maintaining treatment for at least 14 days after the first negative blood culture^31,32^. However, in our series, 68% (15/22) of patients died before completing 14 days of therapy after diagnosis. Another relevant aspect is the absence of echinocandins in our hospital. These agents are not available as initial therapy, as recommended by current guidelines^15,26-28^, which may have contributed to the higher mortality observed in our study.

*C. albicans* was the most prevalent species, followed by *C. parapsilosis* as the leading non-*albicans* species, in agreement with global epidemiology^33^. No resistance was detected among *C. albicans*; only one isolate was classified as SDD to FCZ. *C. parapsilosis* is frequently implicated in HAIs, with healthcare workers or contaminated medical devices described as potential sources of infection, particularly in intensive care settings and during invasive procedures^34,35^. Its ability to form biofilms on medical devices enhances persistence and contributes to reduced susceptibility to antifungal treatments, posing therapeutic challenges and potentially resulting in adverse clinical outcomes^34,35^. Notably, we identified one *C. parapsilosis* isolate resistant to two of the three antifungal classes tested (azoles and echinocandins). This resistant isolate was recovered in 2023, indicating a possible emergence of resistance in this pathogen at our hospital, consistent with global trends^10^.

Additionally, the overall resistance rate of 13% to at least one of the three antifungals tested (FCZ, AMB, and MICA) observed in our study is slightly higher than rates reported in other studies conducted in Brazil and, particularly, in developed countries^22,36,37^. The increasing trend of resistance among *Candida* species has been associated with higher overall mortality^38^. Furthermore, widespread azole prophylaxis has been linked to an increase in infections caused by intrinsically resistant species (e.g., *P. kudriavzevii*) or less susceptible species, particularly *N. glabratus*, representing a global concern^2,33,39^. In our study, these two species were identified, along with one *C. tropicalis* isolate resistant to MICA and one *C. duobushaemulloni* isolate resistant to AMB. Fortunately, these resistant isolates represented a small proportion compared with *C. albicans* and *C. parapsilosis*.

## CONCLUSION

This study highlights the substantial impact of candidemia among ICU patients, with notably high mortality rates. Effective prevention strategies must include rigorous training and surveillance of healthcare staff regarding infection control protocols, proactive monitoring to enable early diagnosis, and implementation of antifungal prophylaxis protocols to reduce the impact of candidemia and improve patient care and safety. These measures are especially critical in our NICU, given the high incidence observed.

## Data Availability

The authors will not make additional data available due to ethical restrictions related to patient confidentiality.
